# Understanding the factors influencing quality of life among survivors of Non-Hodgkin lymphoma after completing primary treatment: a systematic review

**DOI:** 10.1007/s00520-026-10488-2

**Published:** 2026-03-03

**Authors:** Pichitra Lekdamrongkul, Suebsarn Ruksakulpiwat, Jinsuta Tadsuan, Kanaungnit Pongthavornkamol, Alex Molassiotis

**Affiliations:** 1 Department of Medical Nursing, Faculty of Nursing, Mahidol University, Bangkok, Thailand; 2 School of Nursing, College of Medicine, National Taiwan University, Taipei, Taiwan; 3 School of Psychology, Health, and Clinical Sciences, Aston University, Birmingham, UK

**Keywords:** Non-Hodgkin lymphoma, Cancer survivors, Quality of life, Factors

## Abstract

**Purpose:**

To evaluate and synthesize the existing evidence on factors influencing the quality of life (QoL) of non-Hodgkin lymphoma (NHL) survivors and the impact of these factors on their QoL.

**Methods:**

A systematic review was conducted following PRISMA guidelines, with searches in CINAHL, MEDLINE, PubMed, ScienceDirect, Scopus, and Web of Science. Studies published between 2014 and 2025 were included if they were original English-language research involving adult (age ≥ 18 years) NHL survivors and focused on factors affecting QoL. Exclusion criteria encompassed animal studies and nonoriginal research. Data synthesis and quality assessment utilized the convergent integrated analysis framework from the Joanna Briggs Institute to identify key themes across studies.

**Results:**

Nineteen studies (*n* = 8322) were included, revealing nine key themes: (1) personal characteristics (e.g., age and gender); (2) clinical characteristics (e.g., time since diagnosis and comorbidities); (3) physical concerns (e.g., fatigue and symptom burden); (4) psychological concerns (e.g., anxiety, depression, and PTG); (5) lifestyle factors (e.g., diet and exercise); (6) sexual health (e.g., satisfaction and erectile dysfunction); (7) economic status (e.g., employment and financial strain); (8) supporting systems (e.g., unmet needs), and (9) area of residence (e.g., rural residence).

**Conclusions:**

This review highlights the multifactorial influences on QoL in NHL survivors, emphasizing the need for integrated survivorship care that addresses physical, psychological, and social dimensions to improve long-term outcomes. Healthcare providers should prioritize individualized care plans addressing both physical and psychosocial challenges, with digital health interventions, especially for rural populations, to enhance QoL outcomes.

**Supplementary Information:**

The online version contains supplementary material available at 10.1007/s00520-026-10488-2.

## Introduction

Non-Hodgkin lymphoma (NHL) is one of the most common cancers globally, particularly hematologic malignancies. In 2020, there were approximately 545,000 new cases of NHL and 260,000 related deaths worldwide, and the estimated number of new cases of NHL is expected to increase by 42.8% globally from 2020 to 2040 [1]. However, with early detection and advances in medical treatment, the number of cancer survivors has been increasing and becoming a large proportion of the cancer population [2]. In particular, the five-year relative survival rates of NHL are increasing across several countries worldwide [3–5].

According to the National Coalition for Cancer Survivorship (NCCS) of the U.S., a cancer survivor is defined as a person who has been diagnosed and lived with, through and beyond a cancer diagnosis [6]. Mullan [7] described the cancer survivorship trajectory in the following three phases: (1) acute survival (diagnosis to the end of primary treatment); (2) extended survival (transition period from treatment to post-treatment); and (3) permanent survival (long-term survival period). All of these phases cover a cancer survivorship continuum. Past research has revealed that post-treatment NHL survivors are faced with adverse short- and long-term effects induced by different treatments and the specific characteristics of NHL subtypes, which are unique and show several differences from other types of cancer [8–10]. Therefore, the adverse short- and long-term effects resulting from treatment and disease affect NHL survivors’ quality of life (QoL) [10], which is the outcome of cancer survivors indicated by favorable lifestyles and living conditions in this group.

Current research on QoL in NHL indicates that multiple factors affect QoL. Amatya et al. [11] examined the QoL in patients with NHL. The study indicated that individuals with NHL experience physical and psychological symptoms, which adversely affect their QoL [11]. Consistent with Wasse et al. [12] have demonstrated that the QoL of NHL survivors is worse compared to that of people in general, with anxiety and depression leading to a decrease in QoL. Furthermore, NHL survivors’ QoL decreased due to physical [13] and psychological symptoms [14], as well as unmet supportive care needs [15, 16].

Existing research indicates that while numerous studies have examined QoL among NHL patients, only a limited number have concentrated specifically on NHL survivors’ post-primary treatment, particularly regarding the short- and long-term effects and the factors affecting QoL in this demographic. In addition, the findings from this systematic review can contribute to understanding the factors associated with QoL and how to develop programs, interventions, or systems to enhance the QoL in the physical, psychological, emotional, and functional dimensions of NHL survivors after the completion of primary treatment. The authors conducted a comprehensive review and synthesized published studies focusing on the factors associated with QoL in NHL survivors after the completion of primary treatment defined as the first treatment of a disease or part of a standard set of treatments concerning extended and permanent survival phases as described by Mullan [7].

### Objective

The objective was to synthesize the existing evidence to comprehensively understand the factors affecting the quality of life among non-Hodgkin lymphoma survivors after the completion of primary treatment.

## Methods

### Identify relevant studies

This systematic review followed the Preferred Reporting Items for Systematic Reviews and Meta-Analyses (PRISMA) guidelines [17] to delineate the process of literature identification, screening, exclusion, and inclusion. In May 2024, the researchers systematically searched six electronic databases: CINAHL, MEDLINE, PubMed, ScienceDirect, Scopus, and Web of Science, to identify relevant studies published between 2014 and 2024. To ensure the currency of the review, search results were updated in December 2025 to identify any additional eligible studies published prior to publication. This study aimed to explore factors influencing the QoL among NHL survivors after the completion of primary treatment. The study employed Boolean phrases (i.e., AND and OR) to combine search terms such as “hematologic*,” “haematologic*,” “lymphoma*,” “Non-Hodgkin lymphoma*,” “Non-Hodgkin lymphoma survivors,” “quality of life,” “factors,” “physical well-being,” “psychological well-being,” “emotional well-being,” and “functional well-being.” Furthermore, the researchers manually reviewed the reference lists of the included studies to identify any additional relevant material. The researchers managed all the identified references for subsequent analysis using EndNote.

### Study selection

The study selection process followed the PRISMA flowchart. Two reviewers conducted a systematic electronic search of databases to select the studies independently. Initially, titles and abstracts were screened to identify studies that might meet the eligibility criteria. Following this, the full texts of the selected studies were evaluated to confirm their relevance to the review’s objectives. The researchers then applied inclusion criteria to ensure that only studies pertinent to our research question were retained, while exclusion criteria were used to discard literature that did not align with the scope of the review. The inclusion criteria were (1) aged ≥ 18 years; (2) original quantitative, qualitative, or mixed methods studies; (3) investigate the factors influencing the quality of life among non-Hodgkin lymphoma survivors; (4) included participants who were diagnosed with non-Hodgkin lymphoma after completion of primary treatments; (5) all types of settings are acceptable, including inpatient, outpatient, or home; and (6) described in the English language. The exclusion criteria were as follows: (1) the study did not include the population of interest or concerned animal subjects and (2) conference proceedings, abstracts, review articles, theoretical studies, pilot studies, protocols, dissertations, letters to the editor, opinion (viewpoint), statement articles, government documents, or working papers. Both reviewers were required to reach a consensus. Disagreements were resolved by a third independent reviewer, receiving a final decision.

### Quality assessment

The quality assessment aims to examine the methodological rigor of each study and assess how effectively potential biases related to design, conduct, and analysis have been managed. In this review, two independent researchers assessed the methodological quality of the included studies using the Joanna Briggs Institute (JBI) critical appraisal tools, which are specifically designed for systematic reviews [18].

### Data extraction

The standardized chart for data extraction (Supplementary Table 1) developed for this review included the following data for each study: country, references, published year, settings, target population, study design, sample size, age of participants, gender, quality assessment, purpose of study, treatment, time since diagnosis, stage, year of survivor, main outcome (factor impacting QoL), QoL measurement, themes (factor influencing QoL), and implications/suggestions.

### Data synthesis

This study utilized the convergent integrated analysis framework, as recommended by the Joanna Briggs Institute (JBI) for systematic review, to synthesize data from the included studies. The process involved identifying key themes by analyzing similarities and differences within the main findings. In addition, subthemes were developed to highlight more specific aspects of the data, following an approach similar to thematic analysis used in quality research [19].

## Results

### Search results

**Fig. 1:**
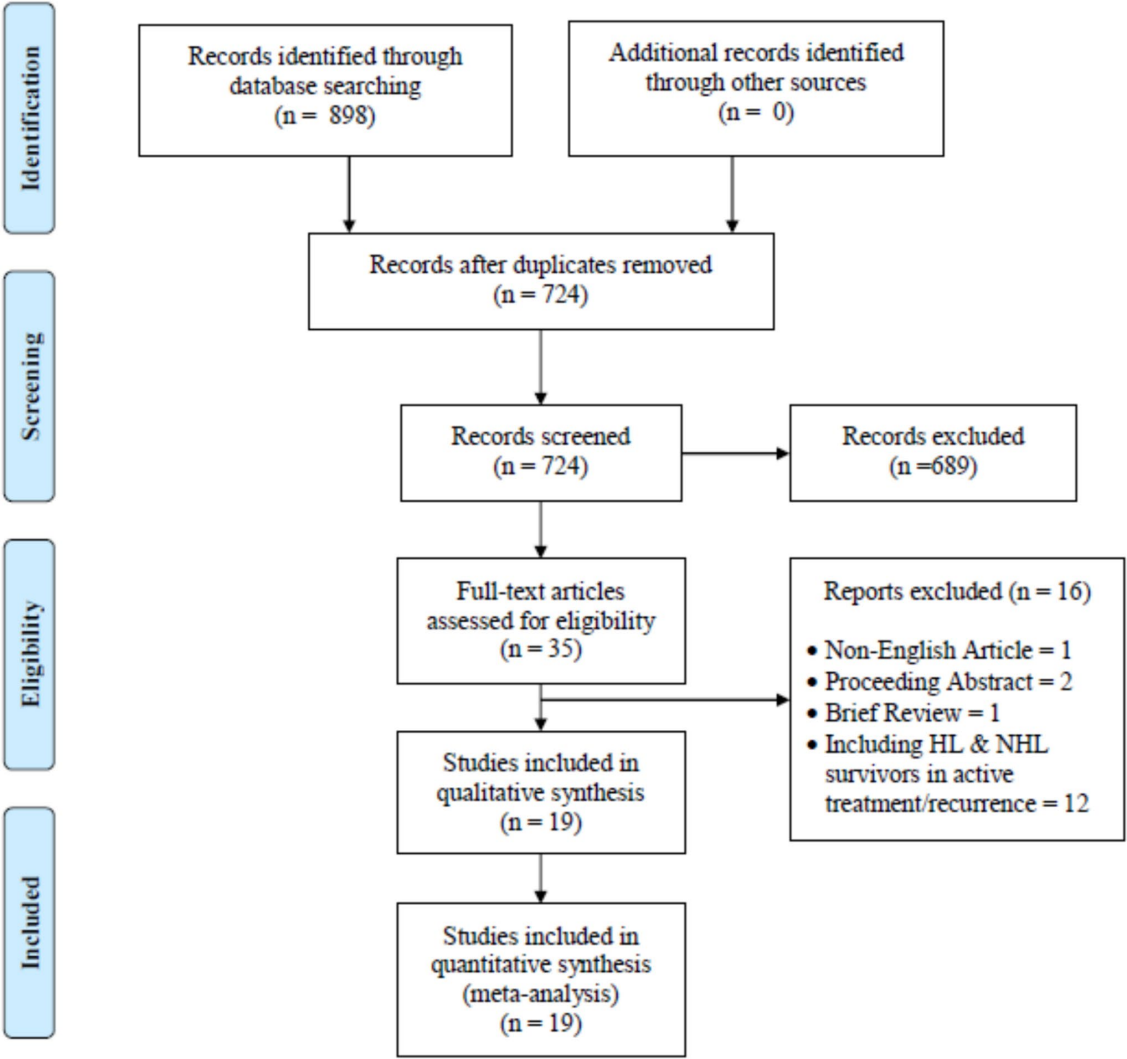
PRISMA flow chart

In this systematic review, we initially identified a total of 898 studies. After 174 duplicates were removed, 724 studies remained for screening based on titles and abstracts following the inclusion and exclusion criteria. During this process, 689 studies were deemed ineligible and subsequently eliminated, leaving 35 studies for full-text assessment. After further investigation, 16 studies were removed for various reasons, including a non-English study (*n* = 1), proceeding abstracts (*n* = 2), a brief review (*n* = 1), and HL and NHL survivors who were still receiving treatment or experiencing recurrence (*n* = 12). Ultimately, the final systematic review included 19 studies, as shown in Fig. 1.

### Characteristics of included studies

Table 1 illustrates the characteristics of the included studies. Most studies were conducted in the USA (26.3%, *n* = 5), followed by South Korea and France (15.8%, *n* = 3). The Netherlands contributed 10.5% (*n* = 2) of the studies. Other countries included Norway, Sweden, Australia, Thailand, Indonesia, and China, each contributing 5.3% (*n* = 1). The studies were published across various years, with the majority from 2022 (15.8%, *n* = 3). Other notable years included 2023, 2021, 2020, 2018, 2017, and 2015, each representing 10.5% (*n* = 2). The remaining studies were published in 2025, 2024, 2016, and 2014, each accounting for 5.3% (*n* = 1).

**Table 1:** Characteristics of included studies

Characteristics	Number of included study	Percentage
Country		
USA	5	26.3
South Korea	3	15.8
France	3	15.8
Netherlands	2	10.5
Norway	1	5.3
Sweden	1	5.3
Australia	1	5.3
Thailand	1	5.3
Indonesia	1	5.3
China	1	5.3
Publication year		
2025	1	5.3
2024	1	5.3
2023	2	10.5
2022	3	15.8
2021	2	10.5
2020	2	10.5
2018	2	10.5
2017	2	10.5
2016	1	5.3
2015	2	10.5
2014	1	5.3
Study setting		
Hospital (not specified)	6	31.6
Postal survey	5	26.3
Clinic (not specified)	2	10.5
Online survey	2	10.5
Electronic health records	2	10.5
Outpatient department	1	5.3
Inpatient department	1	5.3
Target population		
Non-Hodgkin lymphoma (not specified)	8	33.3
Indolent lymphoma	4	16.7
Diffuse large B cell lymphoma (DLBCL)	6	25.0
Aggressive lymphoma	3	12.5
Follicular lymphoma (FL)	2	8.3
Cutaneous T-cell lymphoma (CTCL)	1	4.2
Study design		
Cross-sectional study	17	89.5
Prospective cohort study	1	5.3
Qualitative study	1	5.3
Sample size		
< 200	5	26.3
> 200–400	4	21.1
> 400–800	6	31.6
> 800	4	21.1
Quality-of-life measurement*		
The European Organization for Research and Treatment of Cancer Quality of Life Questionnaire Core 30 (EORTC QLQ-C30)	9	42.9
The Medical Outcomes Study Short Form-36 (SF-36)	4	19.1
Functional Assessment of Cancer Therapy-General, Version 4 (FACT-G)	2	9.5
Functional Assessment of Cancer Therapy-Lymphoma (FACT-LYM)	1	4.8
The Medical Outcomes Study Short Form Health Survey-12 (SF-12)	2	9.5
Euro QOL-5D	1	4.8
The European Organization for Research and Treatment of Cancer Quality of Life Questionnaire-Non-Hodgkin Lymphoma High-Grade 29 (EORTC QLQ-NHL-HG29)	1	4.8
The 5-Leve EQ-5D	1	4.8

***Note.*** One study may report more than one characteristic, hence the total number of included studies can be more than 19

*One included study [20] used a qualitative research design rather than a standardized quality-of-life measurement; therefore, it was not included in the table. *Percentages may not sum to 100% due to rounding*

Study settings were diverse. Most studies were conducted in unspecified hospitals (31.6%, *n* = 6). Other settings included postal surveys (26.3%, *n* = 5), unspecified clinics (10.5%, *n* = 2), online surveys (10.5%, *n* = 2), electronic health records (10.5%, *n* = 2), outpatient departments (5.3%, *n* = 1), and inpatient departments (5.3%, *n* = 1). In terms of study design, cross-sectional studies were predominant (89.5%, *n* = 17), with a few using prospective cohorts (5.3%, *n* = 1) and qualitative designs (5.3%, *n* = 1). Sample sizes varied, with studies equally distributed among 1–200 participants (26.3%, *n* = 5) and > 400–800 participants (31.6%, *n* = 6). Studies with sample sizes > 200–400 (21.1%, *n* = 4) and > 800 (21.1%, *n* = 4) were also represented.

Target populations included a variety of non-Hodgkin lymphoma (NHL) subtypes. The most common target was non-Hodgkin lymphoma (not specified subtypes) (33.3%, *n* = 8). Other subtypes of NHL included indolent lymphoma (16.7%, *n* = 4), diffuse large B cell lymphoma (DLBCL) (25.0%, *n* = 6), aggressive lymphoma (12.5%, *n* = 3), follicular lymphoma (FL) (8.3%, *n* = 2), and less frequently, cutaneous T-cell lymphoma (CTCL) (4.2%, *n* = 1). Regarding quality-of-life measurement tools, the EORTC QLQ-C30 was the most frequently used (42.9%, *n* = 9). Other tools included the SF-36 (19.1%, *n* = 4). Less commonly used were the FACT-G and SF-12 (9.5%, *n* = 2), and less frequently FACT-LYM, Euro QOL-5D, EORTC QLQ-NHL-HG29, and 5-Level EQ-5D (4.8% each, *n* = 1). The majority treatment for participants in this review is chemotherapy, which included targeted therapy. All participants in the reviews were declared at the end of the primary treatment period, which included NHL survivors from stages I to IV; however, a few studies did not specify the staging of the participants.

### Assessment of methodological quality

To ensure the review’s robustness, two independent reviewers rigorously assessed the methodological quality of the included studies using the Joanna Briggs Institute (JBI) critical appraisal checklist specifically designed for systematic reviews [18]. The findings indicate the included studies presented the methodological quality components with clarity; 14 studies were assessed as high quality (100%) [12, 20–30, 35, 36]. However, five studies demonstrated minor limitations, receiving a quality assessment of very acceptable (87.5%) [14, 31–34], resulting in an overall score of 96.7%. Details on the quality assessment for each study are provided in Supplementary Table 1.

### Description of the factors influencing QoL

**Fig. 2:**
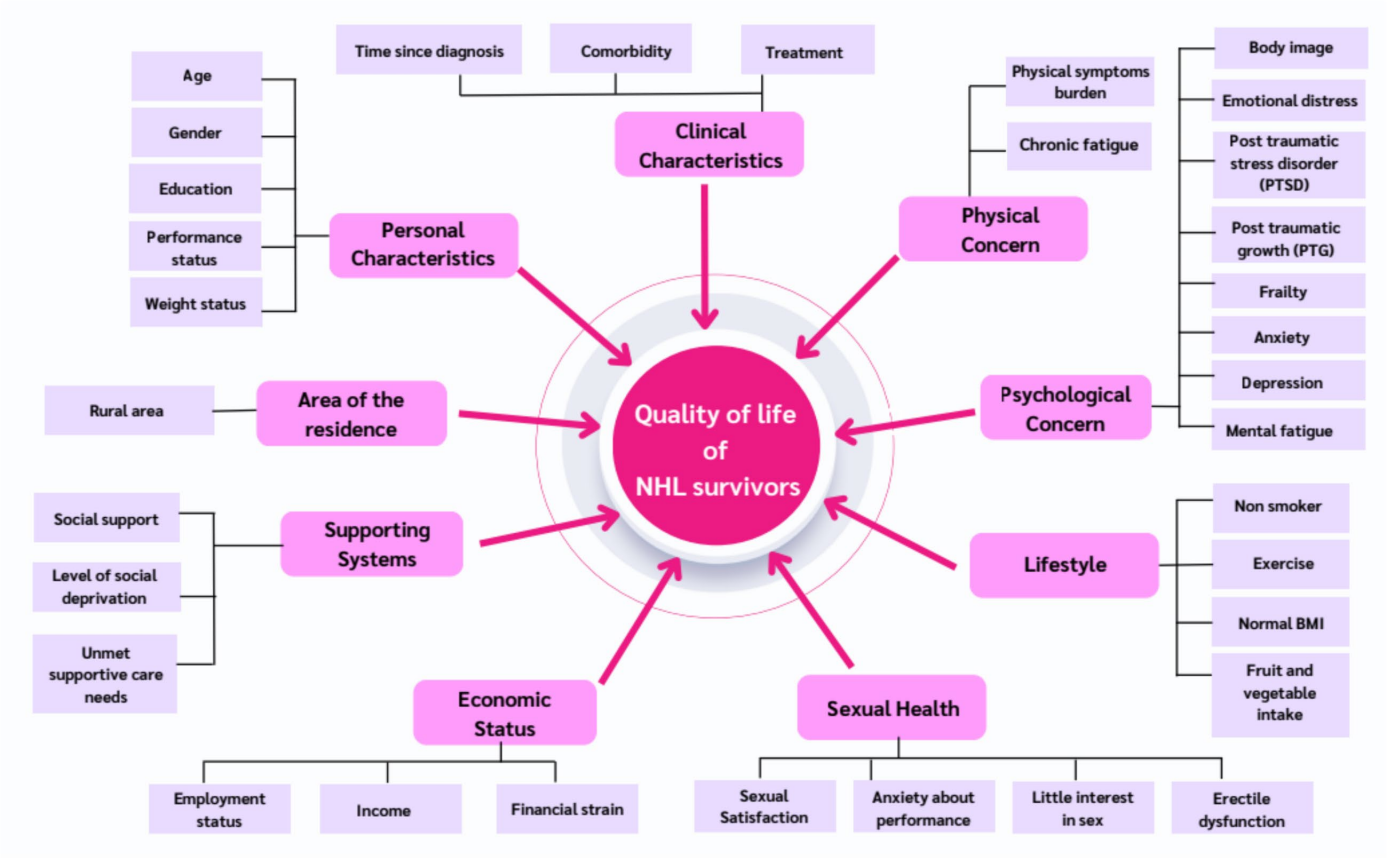
Themed factors influencing quality of life among non-Hodgkin lymphoma (NHL) survivors

The factors influencing the QoL in NHL survivors are summarized in Fig. 2, and the overarching themes derived from the systematic review are presented in Table 2.. The following are the nine overarching themes that emerged from the data synthesis: (1) personal characteristics, (2) clinical characteristics, (3) physical concern, (4) psychological concern, (5) lifestyle, (6) sexual health, (7) economic status, (8) supporting system, and (9) area of residence.

**Table 2.** The themes of factors influencing QoL among non-Hodgkin lymphoma (NHL) survivors

References	Factors affecting quality of life among non-Hodgkin lymphoma survivors (themes)								
	Supporting systems	Lifestyle	Sexual health	Economic status	Area of residence	Personal characteristics	Clinical characteristics	Physical concerns	Psychological concerns
[12]	X					X	X		X
[14]				X			X		X
[21]	X			X		X	X		X
[22]	X						X	X	X
[23]				X				X	X
[20]							X		X
[24]						X			X
[25]						X			X
[26]						X			
[27]						X	X	X	X
[28]				X					
[29]			X						X
[30]	X								X
[31]		X							
[32]				X			X	X	X
[33]							X		X
[34]					X				
[35]			X	X		X	X		X
[36]			X				X		X
Number of studies	4 studies (21.1%)	1 study (5.3%)	3 studies (15.8%)	6 studies (31.6%)	1 study (5.3%)	7 studies (36.8%)	10 studies (52.6%)	4 studies (21.1%)	15 studies (78.9%)

### Personal characteristics

Personal characteristics of NHL survivors include age, gender, education, weight status, and performance status, as shown by seven studies [12, 21, 24–27, 35] that were found to impact QoL. A study by Paunescu et al. [27] revealed that gender difference has an impact on QoL, particularly with women showing significantly worse physical functioning in comparison to men. According to age, Bryant et al. [21] investigated 750 NHL survivors in the United States and reported that older NHL survivors had a higher QoL compared to younger survivors. Conversely, other research shows a contrasting relationship between age and QoL. They revealed that NHL survivors of younger age had an improved QoL compared to older NHL survivors [12]. However, the research conducted in the Netherlands [26] among NHL survivors revealed that younger NHL survivors experienced a negative correlation between age and QoL in cognitive and social functioning. On the other hand, older NHL survivors showed a negative association between age and QoL in physical functioning, emotional functioning, global health status, and overall QoL [35]. In terms of anthropometrics, Wasse et al. [35] demonstrated that deviations from normal weight—specifically underweight and obesity—were significantly associated with impaired physical functioning. Furthermore, regarding educational background, survivors with a higher level of education demonstrated significantly better outcomes in the role-emotional dimension [12]. In addition, the research conducted by Ariestine et al. [25] in Indonesia presented evidence that NHL survivors with high-performance status, as measured by ECOG score, had a better QoL [25].

### Clinical characteristics

Clinical characteristics have been identified as a significant determinant affecting the QoL of NHL survivors from ten publications [12, 14, 20–22, 27, 32, 33, 35, 36]. It covers several variables including the staging of NHL, treatment of NHL, comorbidity, and duration after diagnosis. Chemotherapy is the primary therapy for NHL, known to have a negative effect on QoL [22]; especially in high-grade systemic therapy, which also leads to decreased QoL [33]. Furthermore, a qualitative study highlighted that NHL survivors who had CAR T-cell therapy described experiences of lower QoL, particularly in terms of physical functioning, emotional functioning, role functioning, and social functioning during the post-treatment phase. In addition, decreased QoL had negative impacts on NHL survivors with comorbidities [14, 27, 32, 35, 36], especially in The Physical Component Summary (PCS) dimension [32], physical functioning, and global health status [27]. Additionally, NHL survivors who have a long duration of diagnosis have a higher QoL compared to NHL survivors who have a short period following the end of therapy [12].

### Physical concerns

Physical concerns have been identified as a significant determinant of QoL, as demonstrated in four studies [22, 23, 27, 32]. For example, Lekdamrongkul et al. [22] define physical concern as the physical symptom burden experienced by NHL survivors after finishing the main treatment, both in the short term and long term. Confirmation of the research conducted by Paunescu et al. [27] in France revealed that patients experienced physical symptom burdens such as discomfort, dyspnea, and neuropathy, which negatively impacted their QoL. In particular, persistent fatigue demonstrated a negative relationship between the burden of physical symptoms and QoL among survivors of NHL [32]. Similarly, Kang and colleagues’ research [23], which examined the QoL in long-term NHL patients, showed that physical symptoms such as fatigue, insomnia, and appetite loss after treatment had a negative impact on QoL.

### Psychological concerns

Psychological concerns have been identified as an important variable of QoL in fifteen studies [12, 14, 20–25, 27, 29, 30, 32, 33, 35, 36]. Existing research indicates that NHL survivors experiencing emotional discomfort [20] or psychiatric disorders [33] have a negative impact on QoL, particularly anxiety [12, 22, 24, 29, 32, 35] and depression [12]. Regarding anxiety concerns not just related to illness but also include sexual performance [29]. Furthermore, we can classify psychological concerns into two distinct dimensions: growth mindset and psychological burden resulting from negative events, like cancer. According to Bryant et al. [21], NHL survivors in the US who adopt a growth mindset after experiencing a serious illness like cancer and develop post-traumatic growth (PTG) have a strong relationship to improved QoL. In contrast, NHL survivors who have had cancer and subsequently developed post-traumatic stress disorder (PTSD) have demonstrated worse QoL. Similarly, Lekdamrongkul et al. [22] evaluated the effect of PTG and PTSD on QoL in Thailand and found a similar outcome. In addition, the feeling of fear of cancer recurrence (FCR) has been associated with a decrease in the QoL [14]. Furthermore, both aggressive and indolent NHL survivors express dissatisfaction with their lives, including a lack of hope and a sense of purposelessness [23]. Additionally, it was found that negative body image perception among NHL survivors was associated with poorer QoL [36].

### Lifestyle factors

Lifestyle factors have been accepted as significant contributors to the QoL in NHL survivors. This assertion is supported by a study included in the review, which aimed to examine the relationship between lifestyle factors such as physical activity, fruit and vegetable consumption, maintaining a healthy weight, and smoking—and the QoL of NHL survivors [31]. The results of this cross-sectional study, which included 566 NHL survivors, found that several factors significantly impacted both physical and mental health-related QoL. For the Physical Component Summary, key factors contributing to a higher score (better physical QoL) were being a nonsmoker and engaging in at least 150 min of exercise per week. In terms of mental QoL, significant contributors included being a nonsmoker, meeting the physical activity guideline of 150 min of exercise per week, adhering to the “5-A-Day” fruit and vegetable consumption recommendation, and maintaining a BMI below 25 kg/m^2^ [31].

### Sexual health

Issues related to sexuality, encompassing both dysfunction and satisfaction, were identified as significant factors of QoL in NHL survivors by three studies [29, 35, 36]. Specifically, a population-based study by Wasse et al. [35] confirmed that problems with sexual satisfaction were significantly associated with poorer HRQoL in long-term survivors. Similarly, Olsson et al. [36] highlighted the relational aspect of sexuality, revealing that better sexual relationships were positively associated with HRQoL. Regarding specific dysfunctions, this section was further supported by a cross-sectional study involving 738 NHL survivors, which investigated the prevalence of sexual dysfunction, the related variables, and their correlation with QoL in both male and female survivors [29]. In male survivors, those who experienced a lack of interest in sex, anxiety about sexual performance, or erectile dysfunction demonstrated poorer emotional functioning. In addition, males with all sexual dysfunctions, except erectile dysfunction, reported worse global QoL. In female survivors, only anxiety about sexual performance was significantly linked to reduced emotional functioning, and no significant association was found between sexual problems and global QoL in females [29].

### Economic status

The economic status is reported to strongly influence QoL among NHL survivors, as evidenced by six studies [14, 21, 23, 28, 32, 35]. Bryant et al. [21] reported that NHL survivors with an annual income of less than $30,000 reported significantly lower QoL [21]. Similarly, Xu et al. [28] investigated the relationship between financial burden and QoL in large-scale research that included 1549 NHL survivors in China. The findings showed a significant relationship between financial strain and worse QoL in multiple domains, including EQ-Index, physical, emotional, and social functioning [28]. NHL survivors also reported financial difficulties as a strong influence on decreased QoL [23]. Moreover, NHL survivors report that lacking employment negatively impacts their QoL [14].

### Supporting systems

Supporting systems emerge as a significant factor influencing QoL, as identified in four studies [12, 21, 22, 30]. For instance, Wasse et al. [12], studying survivors of FL and DLBCL, found that satisfaction with social support was significantly associated with improved QoL, particularly in mental and social functioning [12]. Likewise, Kim et al. (2017) [30] found that participants who had unmet supportive care needs in the diet and exercise domain and the support domain exhibited worse role, emotional, social function, and global QoL. Furthermore, unmet supportive care needs in the relationship with healthcare professionals demonstrated worse emotional and social function [30].

### Area of residence

The area of residence has been shown to significantly impact the QoL among NHL survivors. A secondary analysis using data from two large academic medical centers compared self-reported QoL, measured using the SF-36, between rural and nonrural NHL survivors and examined the association between rural status and QoL in NHL survivors [34]. The study found that rural survivors reported lower mean SF-36 Physical Component scores compared to nonrural survivors, while there were no significant differences in the Mental Component scores between the two groups. In addition, rural residence was significantly associated with lower scores in the SF-36 Physical Component and poorer physical functioning [34].

Based on the synthesis of the included studies, the factors influencing QoL throughout the survivorship continuum were categorized into three primary themes: physical, psychological, and lifestyle domains. Table 3 illustrates the evolution of these QoL themes as survivors transition from the extended to the permanent phase. In the physical domain, symptoms shifted from acute post-treatment issues to chronic challenges, such as persistent fatigue. Psychologically, while early burdens like anxiety and fear of recurrence tended to subside over time, depression and social isolation often persisted. In addition, lifestyle priorities changed significantly; the initial focus on adopting healthy behaviors gave way to pressing socioeconomic concerns, with financial strain and employment issues becoming more prominent in the later stages.

**Table 3:** Themed analysis grouped by survivorship phase and quality of life themes

Survivorship phase	Theme	Studies/insights
Extended survival	Physical	Persistent fatigue, neuropathy, dyspnea, insomnia noted soon after treatment completion
	Psychological	Anxiety, depression, fear of recurrence, PTSD more prevalent in early post-treatment phase
	Lifestyle	Early adoption of exercise, diet changes, and smoking cessation linked to better QoL
Permanent survival	Physical	Chronic fatigue and late effects (neuropathy and discomfort) remain significant year later
	Psychological	Anxiety and fear of recurrence tend to decrease over time, but depression and social isolation persist
	Lifestyle	Long-term maintenance of healthy behaviors critical; financial strain and employment issues become more prominent

PTSD, post-traumatic stress disorder

## Discussion

This systematic review synthesizes multidimensional determinants of quality of life (QoL) among survivors of non-Hodgkin lymphoma (NHL) after completion of primary treatment. In keeping with the National Coalition for Cancer Survivorship (NCCS) definition—survivorship as living *with, through, and beyond* a cancer diagnosis—and Mullan’s survivorship trajectory (acute, extended, and permanent phases) [6, 7], our findings are best interpreted through established frameworks that explain *why* specific factors shape QoL and *how* their influence evolves over time. In particular, Lazarus and Folkman’s stress and coping theory (appraisal and coping resources); Engel’s biopsychosocial model (interacting biological, psychological, and social domains); the symptom management theory (symptom experience, management, and outcomes); health behavior change models (e.g., theory of planned behavior and self-determination theory); sexual health frameworks (e.g., PLISSIT); and social support theory (buffering hypothesis) together offer a coherent explanatory scaffold for the patterns observed across studies.

### Personal characteristics

Age, gender, education, performance status, and anthropometric characteristics were consistently associated with QoL in our review [12, 21, 24–27, 35]. From a stress-and-coping perspective, younger survivors often appraise cancer as a greater threat to future roles (education, career, and fertility), which can heighten distress and impair mental QoL; older survivors may possess more developed coping repertoires but face biological decline and multimorbidity that compromise physical functioning—an interaction central to the biopsychosocial view. Gender differences (e.g., poorer physical functioning in women [27]) likely reflect intersecting biological factors (treatment effects and endocrine milieu) and social roles (caregiving and employment). Furthermore, education acts as a crucial coping resource. Survivors with higher education reported better emotional outcomes [12], suggesting that educational background may provide the cognitive resources or health literacy necessary to navigate post-treatment stressors effectively. Anthropometrically, weight extremes exerted a dual burden: malnutrition depleted physiological reserves, while obesity—often exacerbated by corticosteroid-induced sarcopenia—compounded physical impairment with body image distress. This underscores that QoL emerges from intertwined domains rather than isolated attributes.

### Clinical characteristics

Disease stage, treatment intensity (e.g., high-grade chemotherapy and multidimensional burdens reported in CAR T-cell therapy), comorbidities, and time since diagnosis exerted strong effects on QoL [12, 14, 20–22, 27, 32, 33, 35, 36]. Symptom management theory explains these associations: more intensive therapy increases the *symptom experience* (fatigue and neuropathy), complicates *management demands* (polypharmacy and clinic visits), and can depress *outcomes* (functional limitation and role impairment). Comorbidities add physiologic and behavioral complexity, limiting rehab potential and self-management and thereby worsening physical and global health [27, 32]. Conversely, longer time since diagnosis was generally associated with better QoL [12], consistent with adaptation and recalibration of expectations over time (stress appraisal shifts; coping becomes more efficient).

### Physical concerns

Fatigue, neuropathy, dyspnea, insomnia, and appetite loss frequently persisted post-treatment and were closely linked with poorer QoL [22, 23, 27, 32]. The biopsychosocial model predicts that physical limitations restrict participation and social roles, which feed back into mood and self-efficacy. In symptom management terms, fatigue and neuropathy are high-burden symptoms with limited quick fixes; they require multimodal strategies (energy conservation, graded activity, and neuropathic pain management) to improve functional outcomes. These mechanisms explain why even modest symptom relief can translate into meaningful QoL gains.

### Psychological concerns

Anxiety, depression, fear of cancer recurrence (FCR), post-traumatic stress symptoms (PTSS), post-traumatic growth (PTG), and body image were among the most influential correlates of QoL [12, 14, 20–25, 27, 29, 30, 32, 33, 35, 36]. Stress and coping theories clarify that threat appraisals (e.g., FCR and uncertainty) and limited coping resources (financial strain and low support) drive anxiety and depression, reducing mental and social functioning. Compounding this distress, body image disturbances—manifesting as weight deviations [35] or negative physical self-perception [36]—act as persistent “internal threats” to self-identity that further impair QoL. In contrast, PTG reflects positive reappraisal, meaning-making, and enhanced personal strength, which are associated with improved QoL [21, 22]. These dual trajectories—burden vs. growth—highlight the importance of routine distress screening that includes body image assessment and timely psychosocial interventions (CBT, mindfulness, and meaning-centered therapy), with attention to phase-specific needs.

### Lifestyle factors

Adherence to physical activity, healthy diet, weight management, and smoking cessation was linked to better physical and mental QoL [31]. Health behavior change theories explain that intention, perceived control, autonomous motivation, and supportive environments increase the likelihood of *initiating* and *sustaining* healthy behaviors. Physiologically, exercise improves cardiorespiratory fitness and reduces cancer-related fatigue; psychologically, it enhances mood and self-efficacy—illustrating biopsychosocial synergy. These mechanisms justify embedding structured exercise and nutrition programs in survivorship pathways, with digital tools to support adherence (particularly in rural settings [34]).

### Sexual health

Sexual dysfunction (e.g., erectile problems, performance anxiety, and loss of interest) was associated with poorer emotional functioning and, in men, lower global QoL [29]. Gender distinctions were evident: male QoL hinged on physical potency and identity, whereas female outcomes reflected performance anxiety and relational dynamics. This confirms that sexual satisfaction and relationship quality [35, 36]—beyond mere function—are critical to well-being. Sexual health frameworks emphasize that sexuality is integral to identity, intimacy, and self-worth; disruptions therefore propagate emotional distress and relationship strain. Normalizing conversations about sexual health, applying stepped care (e.g., PLISSIT), and offering targeted therapies (medical and psychosexual) can mitigate these effects and enhance QoL.

### Economic status

Economic status emerged as a significant determinant of QoL, encompassing income stability and employment [14, 21, 23, 28, 32, 35]. Consistent with the concept of financial toxicity, financial strain acts as a chronic stressor that depletes coping resources. Evidence indicates that low annual income [21] and high financial burden [23, 28] are directly correlated with reduced physical, emotional, and social functioning. This suggests that financial limitations restrict access to supportive care and adaptive strategies necessary for recovery. Furthermore, unemployment [14] compounds this distress; beyond the loss of revenue, the lack of occupational roles disrupts social identity and limits community engagement. Consequently, financial stability functions not merely as a socioeconomic marker, but as a fundamental resource for maintaining holistic well-being.

### Supporting systems and area of residence

Perceived social support and unmet supportive care needs were strongly tied to QoL [12, 21, 22, 30]. The buffering hypothesis posits that support attenuates the impact of stressors on psychological outcomes, while unmet needs amplify distress and role limitations. Rural residence was associated with lower physical QoL [34], plausibly due to access barriers, travel burden, and fewer specialized services—factors that biopsychosocially constrain symptom management and rehabilitation. Telehealth, outreach exercise programs, and health literacy initiatives are therefore pivotal to reduce geographic disparities.

### Survivorship phases, NHL heterogeneity, and models of care

Interpreting our findings through Mullan’s phases clarifies *when* and *how* interventions should be prioritized. In the extended survival phase (post-treatment transition), acute symptom burden and psychological distress (e.g., FCR and anxiety) are pronounced; thus, care models should emphasize intensive symptom control, proactive distress management, and structured transition planning. Our thematic analysis, stratified by survivorship phase and QoL themes, robustly illustrates this longitudinal trajectory. In the physical domain, symptoms shift from acute post-treatment issues to chronic, persistent late effects, notably fatigue and neuropathy. This persistence requires recognition that these sequelae are chronic conditions demanding long-term, multimodal management strategies, consistent with symptom management theory. Psychologically, while early burdens like anxiety and FCR tend to subside over time, reflecting successful adaptation and reappraisal, the sustained presence of depression and social isolation in the permanent phase is critical. In permanent survival (long-term), late effects (chronic fatigue and neuropathy), economic strain, and lifestyle maintenance dominate; care should pivot to chronic disease management, vocational/financial counselling, and sustained behavior change support. Importantly, NHL’s heterogeneity shapes care trajectories: aggressive subtypes (e.g., DLBCL) treated with curative intent often transition to primary care after oncology discharge, requiring robust survivorship care plans and shared-care protocols; indolent subtypes (e.g., FL and CTCL) usually remain within oncology for ongoing monitoring and episodic treatment, reflecting a chronic-care paradigm. Including both cohorts in this review is essential, as each faces distinct survivorship challenges—post-discharge adjustment and late effects in aggressive disease vs. long-term symptom management, uncertainty, and cumulative toxicity in indolent disease [12, 22, 23, 29–34]. This rationale is consistent with the NCCS survivorship definition and ensures our conclusions speak to the full spectrum of NHL survivorship [6, 7].

### Implications for practice

Grounded in the above mechanisms, survivorship programs should be phase-specific and subtype-sensitive. In extended survival, deploy stepped psychological care (screen-and-treat pathways), comprehensive symptom clinics, and coordinated oncology-primary care handovers. In permanent survival, implement long-term fatigue and neuropathy management, community-based exercise and nutrition programs, financial navigation, and tele-enabled follow-up—particularly for rural survivors [34]. Routine assessment of unmet needs, sexual health, and comorbidity management should be embedded across phases. Health-system integration (shared EHRs, survivorship care plans, and referral protocols) and policy support (coverage for rehabilitation and psychosocial services) are key to translating these learnings into equitable, scalable care.

### Strengths, limitations, and future directions

A strength of this review is its comprehensive synthesis across biological, psychological, social, and environmental determinants, interpreted through robust theories. However, many included studies were cross-sectional and did not stratify outcomes by survivorship phase or precise time since treatment, limiting formal trajectory analyses. Future research should adopt longitudinal designs, explicitly categorize survivors into extended vs. permanent phases, and test mechanism-based interventions (e.g., CBT for FCR in extended survival; multimodal fatigue programs in permanent survival). Such work will refine phase-specific recommendations and strengthen implementation across diverse health system contexts.

## Conclusion

QoL of NHL survivors during the post-treatment phase is defined by the dynamic interaction between enduring clinical sequelae and psychosocial resilience. This synthesis establishes that QoL impairment is primarily driven by chronic treatment-related toxicities, such as fatigue and neuropathy, and heightened psychological distress (e.g., fear of cancer recurrence (FCR) and depression). These symptoms operate mechanistically: physical limitations impair performance status while FCR heightens stress appraisals, directly validating the biopsychosocial and stress/coping frameworks. Optimal survivorship care therefore demands a phase-specific and systemically integrated model: (1) extended survival requires proactive, stepped psychological care and intensive symptom control to manage acute distress and (2) permanent survival necessitates a strategic shift toward chronic disease management of late effects, sustainable lifestyle intervention, and mitigation of systemic care barriers. Ultimately, maximizing QoL requires moving beyond episodic follow-up. It mandates the implementation of multidisciplinary, integrated survivorship pathways that prioritize comprehensive screening for distress and late effects, ensuring timely access to evidence-based behavioral and psychosocial interventions.

## Supplementary Information

Below is the link to the electronic supplementary material.

## Supplementary Information

Below is the link to the electronic supplementary material.

ESM 1 (PDF 185 KB)

ESM 2 (PDF 16.3 KB)

ESM 3 (PDF 30.9 KB)

ESM 4 (PDF 464 KB)

## Data Availability

The datasets analyzed during the current study were derived from publicly available sources (original research articles). All extracted data are included in this published article and its supplementary information files.
