# Supplementary material for: Understanding the factors influencing quality of life among survivors of Non-Hodgkin lymphoma after completing primary treatment: a systematic review
Source: Support Care Cancer. 2026 Mar 3;34(3):266. doi: 10.1007/s00520-026-10488-2 (PMC12957100; doi:10.1007/s00520-026-10488-2)
Supplement: Supplementary file 1 — (PDF 185 KB) [file 520_2026_10488_MOESM1_ESM.pdf]

MAHIDOL UNIVERSITY

Since 1888

No conflict of interest document

Dear Editors: Supportive Care in Cancer

I am enclosing here with a manuscript entitled **“Understanding the factors influencing quality of life among survivors of Non-Hodgkins’ Lymphoma after completing primary treatment: A systematic review”** for publication in "*Supportive Care in Cancer*".

The research project was conducted by our research team:

1) Pichitra Lekdamrongkul, PhD, Deaprtment of Medical Nursing, Faculty of Nursing, Mahidol University, Bangkok, Thailand
2) Suebsarn Ruksakulpiwat, PhD, Deaprtment of Medical Nursing, Faculty of Nursing, Mahidol University, Bangkok, Thailand
3) Jinsuta Tadsuan, PhD, Deaprtment of Medical Nursing, Faculty of Nursing, Mahidol University, Bangkok, Thailand, and School of Nursing, College of Medicine, National Taiwan University, Taipei, Taiwan
4) Kanaungnit Pongthavornkamol, PhD, Deaprtment of Medical Nursing, Faculty of Nursing, Mahidol University, Bangkok, Thailand
5) Alex Molassiotis, PhD, School of Psychology, Health, and Clinical Science, Aston University, Birmingham, UK

This written manuscript has been approved by our research team. All team members have confirmed that they do not have any conflicts of interest related to the manuscript or the study we conducted. All team members have signed the attached copyright document.

Pichitra Lekdamrongkul

Date 07/03/2025

Pichitra Lekdamrongkul

Date 07/03/2025

Suebsarn Ruksakulpiwat

Jinsuta Tadsuan

Date 07/03/2025

Jinsuta Tadsuan

Date 07/03/2025

Kanaungnit Pongthavornkamol

Date 07/03/2025

Alex Molassiotis
