# Supplementary material for: Understanding the factors influencing quality of life among survivors of Non-Hodgkin lymphoma after completing primary treatment: a systematic review
Source: Support Care Cancer. 2026 Mar 3;34(3):266. doi: 10.1007/s00520-026-10488-2 (PMC12957100; doi:10.1007/s00520-026-10488-2)
Supplement: Supplementary file 4 — (PDF 464 KB) [file 520_2026_10488_MOESM4_ESM.pdf]

Supplementary Table 1. Summary Table 19 Studies

Study No./ Quality Assessment	References, Published year, Country, Settings	Target population, Study design, Sample size (n), Age of participants (mean ±SD), Gender	Purpose of Study	Treatment, Time since diagnosis, Stage, Year of Survivor	Main Outcome (Factor Impacting Quality of Life, QoL) Only significant (B, OR, P-value)	QoL Measurement	Themes (factor influencing QoL)	Implications/ Suggestions
**Study 12/100%**	(Wasse et al., 2023)<br><br>2023<br><br>France<br><br>Postal survey (sending the questionnaire to pariticipants based on the information from the cancer registry specialized in hematological malignancies in Cote d'Or area)	- Follicular lymphoma (FL) and Diffuse Large B-cell Lymphoma (DLBCL)<br>- Cross-sectional study<br>- n = 157 (FL = 80, DLBCL = 77)<br>- Age of participants (67±12.4 yrs)<br>- Men 86 (55%)	- To examine the HRQoL of NHL survivors<br>- To examine the association between sociodemographic, clinical characterist-ic, psychological factors (anxiety and depression), and HRQoL of NHL survivors	- CMT, RT, and missing data<br>- 7 yrs (2.3)<br>- Stage I-IV and missing stage<br>- year of survivor: NA	<u>The factors predicting HRQoL focusing on PCS dimension including</u><br>- level of social deprivation (EPICES) ($\beta = -5.1, p = 0.04$)<br><u>The factors predicting HRQoL focusing on MCS dimension including</u><br>- Anxiety ($\beta = -12.1, p = 0.001$)<br>- Depression ($\beta = -16.3, p = 0.001$)<br>- Social Support Satisfaction (SSS) ($\beta = 7.1, p = 0.01$)<br><u>The factors predicting HRQoL in each sclae of SF-12 including</u><br>- General Health (GH) was predicted by anxiety ($\beta = -14.3, p = 0.02$) and EPICES ($\beta = -12.8, p = 0.01$)<br>- Physical functioning scale (PF) was predicted by age at survey ($\beta = -12.1, p = 0.001$)<br>- Mental Health (MH) was predicted by Time since diagnosis (TSDx) ($\beta = 2.7, p = 0.01$), anxiety ($\beta = -27.1, p <$	Short Form Health Survey (SF-12)	- Personal characteristics<br>- Clinical characteristics<br>- Psychological concern<br>- Supporting systems	- HRQoL of FL survivors better than DLBCL survivors therefore the first priority that should be the target group of improving HRQoL is DLBCL survivors<br>- The program for improving HRQoL in PCS and MCS dimension should be adding the decreasing anxiety, depression and level of social deprivation; however, promoting the social support satisfaction can improve the better HRQoL.

Study No./ Quality Assessment	References, Published year, Country, Settings	Target population, Study design, Sample size (n), Age of participants (mean ±SD), Gender	Purpose of Study	Treatment, Time since diagnosis, Stage, Year of Survivor	Main Outcome (Factor Impacting Quality of Life, QoL) Only significant (B, OR, P-value)	QoL Measurement	Themes (factor influencing QoL)	Implications/ Suggestions
					0.0001), and SSS ($\beta = 17.3, p = 0.001$)<br>- Role physical (RP) was predicted by depression ($\beta = -37.2, p < 0.01$)<br>- Role emotional (RE) was predicted by education level ($\beta = 14.1, p < 0.01$), anxiety ($\beta = -18.7, p = 0.01$), depression ($\beta = -48.8, p < 0.001$), and SSS ($\beta = 23.8, p < 0.001$)<br>- Bodily pain (BP) was predicted by staging (III-IV) ($\beta = 25.3, p = 0.006$), and EPICES ($\beta = -21.4, p = 0.01$)<br>- Vitality (VT) was predicted by Sex (men) ($\beta = 12.4, p = 0.04$), cancer relapsed ($\beta = -28.3, p = 0.01$), and depression ($\beta = -36.6, p = 0.002$)<br>- Social functioning (SF) was predicted depression ($\beta = -32.1, p = 0.02$), and SSS ($\beta = 15.7, p = 0.04$)			

Study No./ Quality Assessment	References, Published year, Country, Settings	Target population, Study design, Sample size (n), Age of participants (mean ±SD), Gender	Purpose of Study	Treatment, Time since diagnosis, Stage, Year of Survivor	Main Outcome (Factor Impacting Quality of Life, QoL) Only significant (B, OR, P-value)	QoL Measurement	Themes (factor influencing QoL)	Implications/ Suggestions
**Study 14/**<br><br>**87.5%**	(Ellis et al. 2022)<br>Australia<br>The United States<br>online questionnaire	- NHL diagnosis given at least two-years ago.<br>- Cross sectional study<br>- n = 192<br>- Age range 28–92, (62.7± 10.94)<br>- Male = 100 (52.1%) and female = 92 (47.9%)	- To examine demographics, disease-related factors, and affective states (i.e. stress, anxiety, depression) in regard to QOL and fear of cancer recurrence (FCR) in NHL patients<br>- To examine the correlates of FCR and QOL in NHL patients	- Treatment types: N/A<br>- 68.7% not receiving treatment<br>- 31.3% receiving treatment<br>- Time since diagnosis =6.8 years (SD = 4.94, range: 2–37 years)<br>- stage: NA<br>- year of survivor: NA	Factors predicting lower QOL include:<br>**- Employment status (being unemployed)** β = -0.107, p < 0.05<br>**- Comorbid medical illness** β = 0.113, p < 0.05<br>**- Fear of cancer recurrence** β = -0.159, p < 0.005<br>**- Depression** -0.495, p < 0.005	The Functional Assessment of Cancer Therapy-General (FACT-G, Version 4)	- Clinical characteristics<br>- Psychological concern<br>- Economic status	The findings indicate that addressing depressive symptoms in NHL patients is crucial, as these symptoms may negatively impact their ability to cope with and adjust to the illness.
**Study 20/100%**	(Cheng et al., 2022)<br>2022<br>USA<br>Postal survey (Invitation letter was sent according to the list)	- Diffuse large B-cell lymphoma (DLBCL)<br>- Qualitative study<br>- n = 18<br>- Age range = 33-80 (Mean = 60 years)<br>- Male = 11 (61.1%), Female = 7 (38.9%)	- To explore patient descriptions of key domains of HRQoL in DLBCL patients treated with Chimeric antigen receptor T-cell (CAR T) therapy	- CAR T therapy<br><br>- 6.6 year (mean)<br><br>- NA<br><br>- NA	**Social Functioning:** Patients experienced impairments in their social interactions and activities.<br><br>**Emotional Functioning:** Emotional well-being was affected, with patients reporting increased emotional distress.<br><br>**Fatigue:** Persistent and debilitating tiredness was a significant issue.<br><br>**Physical Functioning:** Physical capabilities and overall physical health were impaired.	NA (Qualitative approach)	- Clinical characteristics<br>- Psychological concern	The findings highlight the importance of comprehensive support for DLBCL patients undergoing CAR T therapy. Healthcare providers should integrate emotional, social, and physical care, and develop targeted interventions for persistent issues like fatigue, sleep disturbances, and pain. Enhanced patient education on potential QoL challenges and coping strategies is essential. Refining patient-reported outcome

Study No./ Quality Assessment	References, Published year, Country, Settings	Target population, Study design, Sample size (n), Age of participants (mean ±SD), Gender	Purpose of Study	Treatment, Time since diagnosis, Stage, Year of Survivor	Main Outcome (Factor Impacting Quality of Life, QoL) Only significant (B, OR, P-value)	QoL Measurement	Themes (factor influencing QoL)	Implications/ Suggestions
					**Cognitive Functioning:** Patients reported difficulties with memory, attention, and other cognitive tasks.<br><br>**Role Functioning:** The ability to fulfill roles at work, home, or in other settings was impacted.<br><br>**Sleep:** Sleep disturbances and poor sleep quality were common.<br><br>**Pain/Discomfort:** Patients experienced pain and discomfort, which persisted even six months after therapy.			measures and ensuring long-term follow-up will further support improved quality of life and better treatment outcomes.
**Study 21/100%**	(Bryant et al., 2015)<br><br>2015<br><br>USA<br><br>Cancer center in university hospital	- NHL survivors (indolent 49.1% and aggressive 45.1%)<br>- Cross-sectional study<br>- n = 750 NHL survivors<br>- Age range = 26-92 (62.4±13.4)<br>- Male 379 (50.5%), female = 371 (49.5%)	- To examine a path model of the personal characteristics with QoL and adaptation as mediated factors	- CMT 79.6%, RT 48.3%, and other<br>- 2-44 yrs (10.3±7.0)<br>- Stage I-IV<br>N/A	- The positive relationship between variables on QoL among NHL survivors are positive adaptation (having the higher score on PTG), old age, and having job (employment status) (p<0.05)<br>- The negative relationship between variables on QoL among NHL survivors are negative adaptation (having PTSD of higher score on PLC), having comorbidities, less social supportold age, having	Functional Assessment of Cancer Therapy-General, version 4 (FACT-G)	- Personal characteristics<br>- Clinical characteristics<br>- Psychological concern<br>- Economic status<br>- Supporting systems	- The intervention for promoting HRQoL among NHL survivors should be focusing on improving the mind set of positive adaptation and decreasing the negative adaptation that can promoted the better HRQoL<br>Moreover, the intervention should be target on NHL survivors who are younger age,

Study No./ Quality Assessment	References, Published year, Country, Settings	Target population, Study design, Sample size (n), Age of participants (mean ±SD), Gender	Purpose of Study	Treatment, Time since diagnosis, Stage, Year of Survivor	Main Outcome (Factor Impacting Quality of Life, QoL) Only significant (B, OR, P-value)	QoL Measurement	Themes (factor influencing QoL)	Implications/ Suggestions
					job (employment status) (p<0.05)			having comorbidities, less social support and didn't have a job
**Study 22/** **100%**	(Lekdamrongkul et al., 2021)<br>2021<br>Thailand<br>Hematology Outpatient Department of 2 university hospital plus postal survey	- Non-Hodgkin lymphoma (NHL)Survivors after completion of primary treatment<br>- Cross-sectional study<br>- n = 312<br>- Age range =18-89 (58.55±14.67)<br>- Female (172 / 55.1%), Male (140 / 44.9%)	- To determine Health-Related Quality of Life (HRQoL) for overall period and different time points<br>- To identify the factors predicting HR-QoL of NHL survivor after completion of primary treatment	- All of the participants completed primary treatment (CMT)<br>- From ≥ 6 mths to ≥ 10 yrs<br>- Stage I-IV<br>- From end of treatment to more than 10 yrs	- Physical symptom distress (p-value 0.001), anxiety (p-value 0.008),, depression (p-value 0.001), and unmet supportive care needs (p-value 0.001), were found to be reliable predictors of health-related quality of life (HR-QoL)<br>- Poor adaptation (low PTG, and high PTSD (p-value 0.0014),) and chemotherapy treatment (p-value < 0.001) were associated with a decrease in HR-QoL.<br>- NHL survivors experienced the lowest HR-QoL between the end of treatment and 6 months after treatment completion, particularly in the domains of physical well-being and functional well-being.<br>- The HR-QoL (Health-Related Quality of Life) of survivors with phase I NHL survivors had the lowest score in the Lym (Lymphoma) domain.	Functional Assessment of Cancer Therapy-Lymphoma (FACT-LYM)	- Clinical characteristics<br>- Physical concern<br>- Psychological concern<br>- Economic status<br>- Supporting systems	- The survivorship care plan should focus on NHL survivors throughout the disease's trajectory, with a particular emphasis on the six months following the end of treatment.<br>- A physical and psychological support program should be established to improve the HRQoL and reduce symptoms based on the needs of survivors.<br>- It is essential to encourage positive adaptation in terms of good perception of life and relieve psychological distress.

Study No./ Quality Assessment	References, Published year, Country, Settings	Target population, Study design, Sample size (n), Age of participants (mean ±SD), Gender	Purpose of Study	Treatment, Time since diagnosis, Stage, Year of Survivor	Main Outcome (Factor Impacting Quality of Life, QoL) Only significant (B, OR, P-value)	QoL Measurement	Themes (factor influencing QoL)	Implications/ Suggestions
**Study 23/100%**	(Kang et al., 2018)<br><br>2018<br><br>South Korea<br><br>Samsung Medical Center (Hospital)	- Indolent and aggressive non-Hodgkin lymphoma<br>- A Prospective Cohort Study<br>- n=370<br>- Age range 18 – 82, (Median age = 51 years)<br>- Male = 205 (55.5%), Female =165 (44.5%)	To evaluate HRQoL in long-term survivors of indolent and aggressive non-Hodgkin lymphoma (NHL)	- Chemotherapy, Radiation therapy,<br>- Surgery, Autologous stem cell transplantation<br>- At least 3 years since diagnosis<br>- NA<br>- NA	**Fear of Relapse and Second Malignancy**: Prevalent among all survivors, regardless of disease aggressiveness or stage.<br><br>**Psychosocial Well-being**: 50% of survivors reported impairments, regardless of NHL type or stage.<br><br>**Support Systems**: Over 65% felt they did not receive sufficient support; financial difficulties at diagnosis were linked to feeling unsupported.<br><br>**Physical and Cognitive Functioning at Diagnosis**: Impaired functioning was significantly associated with a lack of life purpose in long-term survivors.	The European Organization for Research and Treatment of Cancer Quality-of-Life Questionnaire Core 30 (EORTC QLQ-C30)	- Physical concern<br>- Psychological concern<br>- Economic status	- Regular psychological support is crucial for addressing fears of relapse and improving mental well-being in NHL survivors.<br>- Targeted interventions are needed to support mental health and social integration while strengthening support systems and offering financial counseling can alleviate stress.<br>- Integrating routine monitoring and rehabilitation into survivorship care plans is essential to address physical and cognitive issues early.<br>- A comprehensive, multidisciplinary approach is recommended to meet NHL survivors' diverse needs and enhance their long-term quality of life.
**Study 24/100%**	**Drost et al. (2016)**<br>The Netherlands<br>Netherlands Cancer Registry	- lymphoma survivors who were diagnosed with all subtypes of indolent (including chronic	- To examine differences in perceived impact of cancer (IOC) between adolescents	**Treatment**: **Radiotherapy alone**: 9% of adults and elderly; 5% of AYA	AYA lymphoma survivors achieved higher scores on the positive IOC summary scale compared to adult and elderly patients ($p < 0.001$), whereas no significant	- The European Organization for Research and Treatment of Cancer Quality of Life	- Personal characteristics<br>- Psychological concern	- While AYAs tend to have a more positive IOC compared to older survivors, some AYAs also face more negative IOC and may

Study No./ Quality Assessment	References, Published year, Country, Settings	Target population, Study design, Sample size (n), Age of participants (mean ±SD), Gender	Purpose of Study	Treatment, Time since diagnosis, Stage, Year of Survivor	Main Outcome (Factor Impacting Quality of Life, QoL) Only significant (B, OR, P-value)	QoL Measurement	Themes (factor influencing QoL)	Implications/ Suggestions
		lymphocytic leukaemia-like) and aggressive B cell NHL and HL as defined by the International Classification of Diseases for Oncology-3 codes (ICD-O-3) between January 1, 1999, and January 1, 2009<br>- Longitudinal population-based survey (Cross-sectional)<br>- 1,281 eligible→ n=861 completed the questionnaire<br>- Age (at time of diagnosis)<br>- Adolescents and young adults: 28.0 (SD = 5.4)<br>- Adults: 53.4 (SD= 8.0)<br>- Elderly: 71.4 (SD = 4.0)<br>- Adolescents and young adults: 50%male<br>- Adults: 60%male<br>- Elderly: 63%male	and young adults (AYAs; 18–35 years at cancer diagnosis), adults (36–64 years) and elderly (65–84 years) with a history of (non-)Hodgkin lymphoma.<br>- To investigate the association of socio-demographic, clinical and psychological characteristics with IOC; and the association between IOC and health-related quality of life (HRQoL) among AYAs only.	**Chemotherapy alone**: 39% of AYA, 46% of adults, 50% of elderly<br>**Chemotherapy + radiotherapy**: 55% of AYA, 20% of adults, 13% of elderly<br>**Active surveillance**: 24% of adults and 26% of elderly; not applicable to AYA<br><br>- **Time since Diagnosis**:<br>**≤2 years**: 12% of AYA, 14% of adults, 18% of elderly<br>**2–5 years**: 40% of AYA, 38% of adults, 51% of elderly<br>**5–10 years**: 48% of AYA, 46% of adults, 30% of elderly<br>**>10 years**: 2% of adults, 2% of elderly; not applicable to AYA<br>**Stage at Diagnosis**:	differences were found for the negative IOC scale.<br>**Factors impact of cancer (IOC) in AYA (18–35 years)**<br>**- Gender** (Female)appearance concerns (B=-.21, p < .05)<br>**- Psychological distress** (anxiety and depression) altruism/empathy B=.28, p<.05; health awareness B=.29, p<.05; appearance concerns B=.31, p<.01; body change concerns B=.4, p<.01; life interferences B=.49, p<.01; worry B=.31, p<.01;<br>**- Marital status** (having a partner) body change concerns (B=-.21, p < .05)<br><br>**impact of cancer (IOC) QoL**<br>**Positive impact**<br>**-Altruism/empathy** (emotional functioning B=−.30, p < .01; cognitive functioning B= -.21, p < .05; global health status B=-.25, p < .05)<br>**-Health awareness** (emotional functioning B=−.31, p < .01; cognitive functioning B= -.30, p < .01;	Questionnaire-Core 30 (EORTC QLQ-C30)<br><br>- Impact of Cancer Scale version 2 (IOCv2)		benefit from interventions tailored to their developmental stage to address their specific concerns.

Study No./ Quality Assessment	References, Published year, Country, Settings	Target population, Study design, Sample size (n), Age of participants (mean ±SD), Gender	Purpose of Study	Treatment, Time since diagnosis, Stage, Year of Survivor	Main Outcome (Factor Impacting Quality of Life, QoL) Only significant (B, OR, P-value)	QoL Measurement	Themes (factor influencing QoL)	Implications/ Suggestions
				**Stage I**: 19% of AYA, 24% of adults, 25% of elderly<br>**Stage II**: 50% of AYA, 18% of adults, 17% of elderly<br>**Stage III**: 19% of AYA, 14% of adults, 12% of elderly<br>**Stage IV**: 10% of AYA, 24% of adults, 23% of elderly<br>**Unknown**: 1% of AYA, 20% of adults, 24% of elderly<br><br>-N/A	social functioning B=-.25, p < .05)<br><br>**Negative impact**<br>**- Appearance concerns** (physical functioning B=-.32, p < .01; emotional functioning B=−.39, p < .01; cognitive functioning B= -.35, p < .01; global health status B=-.37, p < .01)<br>**- Body change concerns** (physical functioning B=-.49, p < .01; role functioning B=−.38, p < .01; emotional functioning B=−.55, p < .01; cognitive functioning B= -.58, p < .01; social functioning B=-.50, p < .01; global health status B=-.59, p < .01)<br>**- Life interferences** (physical functioning B=-.42, p < .01; role functioning B=−.39, p < .01; emotional functioning B=−.57, p < .01; cognitive functioning B= -.64, p < .01; social functioning B=-.61, p < .01; global health status B=-.59, p < .01)<br>**- Worry** (physical functioning B=-.29, p < .01; role functioning B=−.29, p < .05; emotional functioning B=−.42, p < .01; cognitive			

Study No./ Quality Assessment	References, Published year, Country, Settings	Target population, Study design, Sample size (n), Age of participants (mean ±SD), Gender	Purpose of Study	Treatment, Time since diagnosis, Stage, Year of Survivor	Main Outcome (Factor Impacting Quality of Life, QoL) Only significant (B, OR, P-value)	QoL Measurement	Themes (factor influencing QoL)	Implications/ Suggestions
					functioning B= -.37, p < .05; social functioning B=-.31, p < .01; global health status B=-.39, p < .01)			
**Study 25/100%**	**Ariestine et al. (2021)**<br>Indonesia<br>Three public hospitals in Jakarta	- Patients ≥ 60 years, whose been diagnosed with NHL and had received at least four cycles of chemotherapy<br>- Cross sectional study<br>- n=62 participants<br>- Age range = 66-83, mean age: NA, median age = 66<br>- 56.5% male, 43.5% female	To analyze the relationship between factors in geriatric assessment, which are frailty status, nutritional status, functional status, cognitive status, depression status, comorbidity, type of lymphoma, performance status, type of chemotherapy and lymphoma stage with QoL in older patients with NHL	– Treatment types: chemotherapy<br>– Time since diagnosis: NA<br>– Stage: (I = 11.3%, II = 37.1%, III= 12.9%, IV = 35.5%)<br>– Year of survivors: N/A	**SF-36 Physical Component Summary (PCS):**<br>- Depression: PR = 12.086 (95% CI 1.596-92.124), p = 0.016<br>- Frailty: PR = 5.622 (95% CI 1.060-29.807), p = 0.042<br>**SF-36 Mental Component Summary (MCS):**<br>- Depression: PR = 24.400 (95% CI 2.961-140.539), p = 0.016<br>**EORTC QLQ-C30 Functional Scales:**<br>- ECOG Performance Status: PR = 171 (95% CI 8.470-3452.28), p = 0.001	– Short Form-36 Questionnaire (SF-36)<br>– The European Organization for Research and Treatment of Cancer Quality of Life Questionnaire-Core 30 (EORTC QLQ-C30)	- Personal characteristics<br>- Psychological concern	- The findings suggest incorporating comprehensive geriatric assessments, with a focus on depression and frailty screening, into the care of older NHL patients.<br>- Managing depression should be a priority, given its significant impact on both physical and mental quality of life.<br>- Monitoring frailty and using ECOG performance status can help identify potential quality of life issues.<br>- Developing targeted interventions for depression, frailty, and functional status could improve outcomes.

Study No./ Quality Assessment	References, Published year, Country, Settings	Target population, Study design, Sample size (n), Age of participants (mean ±SD), Gender	Purpose of Study	Treatment, Time since diagnosis, Stage, Year of Survivor	Main Outcome (Factor Impacting Quality of Life, QoL) Only significant (B, OR, P-value)	QoL Measurement	Themes (factor influencing QoL)	Implications/ Suggestions
**Study 26/100%**	**van der Poel et al. (2014)** The Netherlands Eindhoven Cancer Registry (ECR), encompassing 2.3 million inhabitants, 18 hospitals, and 2 radiotherapy institutes.	- Diffuse Large B Cell Lymphoma (DLBCL) survivors<br>- Comparative cross-sectional study<br>- n=307 participants<br>- mean age: 63.7 ± 12.9 years for DLBCL survivors 63.5 ± 13.2 years for the normative population<br>- Male = 65%, female = 35% (DLBCL survivors)<br>- Male = 67%, female = 33% (normative population)	– To compare HRQOL between DLBCL survivors of different age categories.<br>– To compare their HRQOL with an age- and sex-matched normative population.	**Treatment:**<br>- Radiotherapy: 18-59 years: 35 (36%), 60-75 years: 39 (27%), 76-85 years: 16 (24%)<br>- Chemotherapy: 18-59 years: 93 (97%), 60-75 years: 135 (93%), 76-85 years: 63 (95%)<br>- Stem cell transplantation: 18-59 years: 3 (3%), 60-75 years: 2 (1%), 76-85 years: 2 (3%)<br>- No therapy: 18-59 years: 1 (1%), 60-75 years: 5 (3%), 76-85 years: 1 (2%)<br>**Years since diagnosis: 3.4 years**<br>- Stage: I 18-59 years 34 (35%), 60-75 years: 51 (35%), 76-85	**Age:** A significant factor affecting various aspects of quality of life<br>- Younger survivors (18-59 years) reported worse cognitive and social functioning (p<0.01)<br>- Older survivors (76-85 years) reported worse physical functioning (p<0.01)<br>**Physical symptoms:**<br>- Dyspnea: More prevalent in younger survivors (p<0.01, medium effect size)<br>- Appetite loss: More prevalent in older survivors (p<0.01)<br>- Constipation: More prevalent in older survivors (p<0.05)<br>**Financial problems:**<br>- More prevalent in younger survivors (p<0.01)<br>**Global health status/overall quality of life:**<br>- Older survivors reported worse outcomes compared to younger survivors (p<0.05)	- The European Organization for Research and Treatment of Cancer Quality of Life Questionnaire-Core 30 (EORTC QLQ-C30)	- Personal<br>- Characteristics	- Younger DLBCL survivors face more significant QoL impairments compared to older survivors, necessitating tailored interventions for their specific challenges.<br>- The worse QoL in older survivors is likely due to age rather than the disease, supporting the use of standard treatments for elderly patients based on their overall health.

Study No./ Quality Assessment	References, Published year, Country, Settings	Target population, Study design, Sample size (n), Age of participants (mean ±SD), Gender	Purpose of Study	Treatment, Time since diagnosis, Stage, Year of Survivor	Main Outcome (Factor Impacting Quality of Life, QoL) Only significant (B, OR, P-value)	QoL Measurement	Themes (factor influencing QoL)	Implications/ Suggestions
				years: 20 (32%)<br>- Stage II: 18-59 years: 24 (25%), 60-75 years: 34 (24%), 76-85 years: 16 (26%)<br>- Stage III: 15-59 years 22(23%), 60-75 years: 27 (19%), 76-85 years: 11 (18%)<br>- Stage IV:18-59 years: 16 (17%), 60-75 years: 32 (22%), 76-85 years: 15 (24%)<br>- Year of survivor: NA				
**Study 27/100%**	**Alexandra-Cristina et al. (2022)**<br><br>France<br><br>Multicenter study conducted in 15 hospitals specializing in the	– diffuse large B-cell lymphoma (DLBCL) patients<br>– Cross sectional study<br>– 101 patients were eligible, with n= 57 participants completing the study	- To investigate HRQoL in DLBCL survivors 1 year after diagnosis, focusing on differences due to gender, comorbidities, and number of	– Treatment types: chemotherapy<br>– Time since diagnosis: 1 year<br>– Stage: I-II = 37.7%, III-IV =62.3%	- **Gender** differences showed that women had significantly lower physical functioning and higher scores for constipation compared to men (Physical functioning: 70.9 vs. 82.1, P = 0.031; Constipation: 27.9 vs. 11.7, P = 0.025).	- The European Organization for Research and Treatment of Cancer Quality of Life Questionnaire-Core 30 (EORTC QLQ-C30)	- Personal characteristics<br>- Clinical characteristics<br>- Physical concern<br>- Psychological concern	- Differences in HRQoL between men and women should be addressed, with a focus on providing tailored supportive care.<br>- Comorbidities significantly impact HRQoL, suggesting

Study No./ Quality Assessment	References, Published year, Country, Settings	Target population, Study design, Sample size (n), Age of participants (mean ±SD), Gender	Purpose of Study	Treatment, Time since diagnosis, Stage, Year of Survivor	Main Outcome (Factor Impacting Quality of Life, QoL) Only significant (B, OR, P-value)	QoL Measurement	Themes (factor influencing QoL)	Implications/ Suggestions
	treatment of hematological cancers	– Mean age ± SD male: 58.7 ±15; range:20-79 years female: 58.2 ±15.8; range: 26-84 years<br>– Male = 54.4%, female = 45.6%	chemotherapy cycles.	– year of survivor: 1 year	- **Presence of comorbidities** was associated with lower global health status and physical functioning, higher symptom burden, and higher scores for depression and mental fatigue (Global health status: 58.9 vs. 73.5, P = 0.017; Physical functioning: 70.4 vs. 82.5, P = 0.035; Symptom burden: 34.2 vs. 18.8, P = 0.021; Depression: 7.7 vs. 4.8, P = 0.019; Mental fatigue: 10.8 vs. 8.2, P = 0.049).<br>- **Patients with more than 6 cycles of chemotherapy** had higher scores for pain, dyspnoea, and neuropathy (Pain: 35.5 vs. 16.6, P = 0.046; Dyspnoea: 36.7 vs. 17.3, P = 0.033; Neuropathy: 44.7 vs. 19.9, P = 0.010).	- The European Organization for Research and Treatment of Cancer Quality of Life Questionnaire-Non-Hodgkin Lymphoma High-Grade 29; (EORTC QLQ-NHL-HG29)		the need for comprehensive management strategies to address physical and psychological health in survivors.<br>- The number of chemotherapy cycles affects certain symptoms, highlighting the importance of monitoring and managing long-term side effects of treatment.
**Study 28/100%**	**Xu et al. 2020**<br>China<br>Nationwide online survey	- Non-Hodgkin lymphomas (NHL)<br>- Cross sectional study<br>- n=1549 participants<br>- Mean age: 43.3 years; SD:NA	To assess the association of HRQoL with financial burden among patients with NHL in China	- Treatment types: chemotherapy, immunotherapy, radiation therapy, and surgery	- **Financial strain:** significantly impacts HRQoL; increased financial burden correlates with reduced HRQoL scores (EQ-Index, physical, emotional, and social	- The 5-Level EQ-5D (EQ-5D-5L)<br>- The European Organization for Research and Treatment of Cancer Quality	- Economic status	- Medical professionals should select highly cost-effective treatments and involve patients in decision-making to ensure they understand the financial

Study No./ Quality Assessment	References, Published year, Country, Settings	Target population, Study design, Sample size (n), Age of participants (mean ±SD), Gender	Purpose of Study	Treatment, Time since diagnosis, Stage, Year of Survivor	Main Outcome (Factor Impacting Quality of Life, QoL) Only significant (B, OR, P-value)	QoL Measurement	Themes (factor influencing QoL)	Implications/ Suggestions
		- Male = 52.2%, Female = 47.8%		- Time since diagnosis: Less than six years for approximately 88% of respondents<br>- Stage: NA<br>- Year of survivor: NA	functioning) in both subjective and objective assessments.<br>- Compared to objective financial burden, subjective financial burden was more strongly associated with worse HRQoL across all domains. Social functioning scores decreased quicker than other HRQoL components, whether measured subjectively (β = -43.6, p < 0.001) or objectively (β = -8.8, p < 0.001).	of Life Questionnaire-Core 30 (EORTC QLQ-C30)		consequences of treatments.<br>- Enhanced social support is critical for improving HRQoL in NHL patients facing financial burdens.<br>- Policy adjustments are needed to better protect patients from the financial impact of NHL treatment.
**Study 29/100%**	**(Kim et al. 2017)**<br>South Korea<br>Three university hospitals	- Non-Hodgkin lymphomas (NHL)<br>- Cross sectional study<br>- n=738 participants<br>- Mean age: 55.3 ± 12.0 years, range 20-89<br>- Male = 57.6%, female = 42.4%	- To examine the prevalence and factors associated with sexual problems and their relationship to HRQOL in male and female NHL survivors	- Treatment types: Chemotherapy (88.8%), Radiation Therapy (RT) on pelvic area (14.8%), Peripheral Blood Stem Cell Transplantation (PBSCT) (10.3%)<br>- Time since diagnosis: Mean 6.2 years	**Sexual problem** are associated with HRQOL.<br>- In male, those who experienced a lack of interest in sex, anxiety about sexual performance, or erectile dysfunction showed poorer emotional functioning (p < .001). Male with all sexual dysfunctions except erectile dysfunction also reported worse global QoL (p < .001).<br>- In female, only anxiety about sexual performance	- EORTC QLQ-C30 (Emotional function and global QoL scales)	- Psychological concern<br>- Sexual health	- The study highlights the need for sex-specific interventions to address sexual problems in NHL survivors, as these issues significantly impact HRQOL, especially in men.<br>- Regular assessment of sexual health should be integrated into routine follow-up care for male survivors.<br>- For female survivors, addressing anxiety about sexual

Study No./ Quality Assessment	References, Published year, Country, Settings	Target population, Study design, Sample size (n), Age of participants (mean ±SD), Gender	Purpose of Study	Treatment, Time since diagnosis, Stage, Year of Survivor	Main Outcome (Factor Impacting Quality of Life, QoL) Only significant (B, OR, P-value)	QoL Measurement	Themes (factor influencing QoL)	Implications/ Suggestions
				(SD 3.1), range 2.1 to 20.9 years<br>- Stage: I-III = 73.2%, IV = 26.8%<br>- year of survivor: NA	was significantly associated with reduced emotional functioning (p < .001). There was no significant link between sexual problem and global QoL in female.			performance may improve emotional functioning, even though it does not significantly affect global QoL.
**Study 30/100%**	**(Kim et al. 2017)**<br>South Korea<br>Three university hospitals	- Non-Hodgkin lymphomas (NHL)<br>- Cross sectional study<br>- n=826 participants<br>- Mean age: 55.6 ± 12.0 years, range 20-89<br>- Male = 58.1%, female = 41.9%	- To describe the prevalence and correlates of unmet needs among NHL survivors in Korea and to identify their association with health-related quality of life (HRQOL).	- Treatment types: 88.5% received chemotherapy, 34.3% received radiation therapy, 10.0% received peripheral blood stem cell transplantation (PBSCT).<br>- Time since diagnosis: Mean 6.3 years (range 2.1–20.9 years).<br>- Stage: I-III = 72.9%, IV = 27.1%<br>- Year of survivor: NA	- Participants who reported unmet needs showed significantly lower levels of functioning and overall quality of life (QOL).<br>- When there were clinically significant differences, those with unmet needs in the diet and exercise domain and the support domain experienced poorer role function , emotional function, social function, and overall QOL (p<0.001).<br>- Similarly, participants with unmet needs in the relationship with health professionals domain exhibited worse emotional function (p<0.001) and social function (p<0.001).<br>- Additionally, those with unmet needs in the treatment and prognosis	- The European Organization for Research and Treatment of Cancer Quality of Life Questionnaire-Core 30 (EORTC QLQ-C30)	- Psychological concern<br>- Supporting systems	- Supportive care programs could enhance HRQOL by addressing unmet needs, particularly in treatment and prognosis, and psychological domains.<br>- Interventions should focus on younger survivors, unmarried individuals, and those with low income to effectively address their unmet needs.

Study No./ Quality Assessment	References, Published year, Country, Settings	Target population, Study design, Sample size (n), Age of participants (mean ±SD), Gender	Purpose of Study	Treatment, Time since diagnosis, Stage, Year of Survivor	Main Outcome (Factor Impacting Quality of Life, QoL) Only significant (B, OR, P-value)	QoL Measurement	Themes (factor influencing QoL)	Implications/ Suggestions
					and keeping mind under control domains had poorer social function (p<0.001). Consequently, unmet needs in all domains were significantly associated with lower social function.<br>- Multivariate logistic analyses indicated that younger age, being unmarried, and low monthly income were associated with unmet needs across multiple domains.			
**Study 31/87.5%**	(Spector et al., 2015)<br>2015<br>USA<br>Postal survey	- NHL survivors<br>- Cross-sectional study<br>- n = 566 NHL survivors<br>- Age of participants (67.2 ±12.5)<br>- Male = 272 (48.1%), female = 294 (51.9%)	- To examine whenever NHL survivors meet the ACS health-related requirements for physical activity, fruit and vegetable intake, healthy weight, and tobacco use for cancer survivors.<br>- To examine the relationship between lifestyle factors and HRQoL of NHL survivors	- Treatment,<br>- 15.2 Yrs (7.2)<br>- Stage<br>- Year of Survivor	<u>The factors are leading to the higher score of PCS dimension including</u><br>- Non-smoker ($\beta = 0.37, p = 0.02$)<br>- Exercise ≥ 150 mins/wk ($\beta = 0.49, p < 0.0001$)<br><u>The factors are leading to better physical HRQoL including</u><br>- Meeting all 4 healthy recommendations ($\beta = 0.57, p < 0.0001$)<br>- Meeting 3 recommendations ($\beta = 0.49, p < 0.0001$)	The Medical Outcomes Study Short Form-36 (SF-36)	- Lifestyle	Promoting the healthy life style as ACS recommendations can improve the HRQoL including PCS and MCS dimension

Study No./ Quality Assessment	References, Published year, Country, Settings	Target population, Study design, Sample size (n), Age of participants (mean ±SD), Gender	Purpose of Study	Treatment, Time since diagnosis, Stage, Year of Survivor	Main Outcome (Factor Impacting Quality of Life, QoL) Only significant (B, OR, P-value)	QoL Measurement	Themes (factor influencing QoL)	Implications/ Suggestions
					The factors are leading to better mental HRQoL including<br>- Non-smoker ($\beta = 0.71, p < 0.0001$)<br>- Meeting physical activity (Exercise ≥ 150 mins/wk ($\beta = 0.41, p < 0.0001$)<br>- 5-A-Day recommendations ($\beta = 0.18, p = 0.03$)<br>- Meeting all 4 healthy recommendations ($\beta = 0.47, p = 0.002$)<br>- BMI <25kg/m^2 ($\beta = -0.21, p = 0.02$)			
**Study 32/ 87.5%**	(Kiserud et al., 2023)<br>2023<br>Norway<br>Two Nordic clinical trials<br>(Sweden 62%)<br>Norway (38%)	- Indolent Non-Hodgkin lymphoma (NHL)(INHL) Survivors after completion of primary treatment and normal population<br>- Cross-sectional study (comparison study)<br>- n=136 participants of INHL<br>- 680 participants of normal population<br>- 23-79 (Median 56 yrs)<br>- Female 75 (55%)	- To determine Health-Related Quality of Life (HRQoL) and fatigue of long term survivors after end of treatment	Stage II-IV and unknown stage	The factors predicting physical HRQoL (PCS dimension) including<br>- Comorbidities (higher number of comorbidities have the lower score of PCS) comorbidities 1-2 [$\beta - -6.1\ p\ Value$ 0.01) and comorbidities 3+ ($\beta - 9.1\ p\ Value <$ 0.001)<br>- Didn't have paid work have lower score of HRQoL in PCS dimension ($\beta - 6.0\ p\ Value$ 0.03)<br>- Having chronic fatigues leading to worst HRQoL	The Short Form Health Survey-36 (SF-36)	- Clinical characteristics<br>- Physical concern<br>- Psychological concern<br>- Economic status	To promote the HRQoL in both dimension (physical and mental) the tailor program of improving HRQoL should be focusing on the NHL survivors who have comorbidities, didn't have paid work, didn't have partner, youger participants, having mental problem as anxiety and depression

Study No./ Quality Assessment	References, Published year, Country, Settings	Target population, Study design, Sample size (n), Age of participants (mean ±SD), Gender	Purpose of Study	Treatment, Time since diagnosis, Stage, Year of Survivor	Main Outcome (Factor Impacting Quality of Life, QoL) Only significant (B, OR, P-value)	QoL Measurement	Themes (factor influencing QoL)	Implications/ Suggestions
					in PCS dimension ($\beta - 10.9\ p\ Value < 0.001$)<br>The factors predicting physical HRQoL (MCS dimension) including<br>- Age at survey (old age of participants having the better HRQoL in MCS dimension) ($\beta = 0.09$, $p\ Value = 0.07$)<br>- Anxiety and depression are predicted HRQoL in MCS dimension) ($\beta = -1.61,\ p\ Value < 0.001;\ \beta = -0.73,\ p\ Value < 0.001$)			
**Study 33/ 87.5%**	(Holahan et al., 2018)<br>2018<br>USA<br>The Multidisciplinary Cutaneous Lymphoma Clinic	- Prospective nonblinded survey<br>- Cross-sectional<br>- n=105<br>- Mean = 61.07 (SD = 13.98)<br>- Male = 68 (64.8%), Female = 37 (35.2%)	To establish baseline HRQoL data for patients with cutaneous T-cell lymphoma (CTCL) and identify its influencing factors	- Skin-directed therapy, Systemic-directed therapy<br>- Time since diagnosis: N/A<br>- Stage I = 88 (83.81%), II-IV = 17 (16.19%)<br>- Year of survivors: N/A	HRQoL in CTCL appears related to a number of factors ($p < 0.05$), including:<br>- Presence of a psychiatric condition (negative impact)<br>- Use of systemic (particularly high grade) therapy (negative impact)<br>- Number of medical comorbidities (negative impact)<br>- Income (negative impact)	The Euro QOL 5D	- Clinical characteristics<br>- Psychological concern	- This study emphasizes the need for a comprehensive approach to care for CTCL patients.<br>- Key suggestions include integrating psychiatric support, personalizing therapy to minimize adverse effects, managing comorbidities effectively, addressing socioeconomic barriers, and providing age-specific care.<br>- These strategies can improve HRQoL for CTCL patients by

Study No./ Quality Assessment	References, Published year, Country, Settings	Target population, Study design, Sample size (n), Age of participants (mean ±SD), Gender	Purpose of Study	Treatment, Time since diagnosis, Stage, Year of Survivor	Main Outcome (Factor Impacting Quality of Life, QoL) Only significant (B, OR, P-value)	QoL Measurement	Themes (factor influencing QoL)	Implications/ Suggestions
								addressing their multifaceted needs. Future research should develop and evaluate interventions targeting these areas.
**Study 34/ 87.5%**	**(Noonan et al., 2020)**<br>The United States<br>Two large academic medical centers	- Non-Hodgkin lymphoma (NHL) Survivors<br>- A secondary analysis<br>- n=566 participants (187=rural, 379=non-rural)<br>- Mean age and range: NA;<br>- Most of participant age≥65 years old (59.9%)<br>- Male = 48.1%, female = 51.9%	- To compare self-reported quality of life and the impact of cancer in rural and non-rural NHL survivors<br>- To examine the association between rural status and QOL in NHL survivors.	- Treatment types: NA<br>- 92.8% not receiving treatment<br>- 7.2% receiving treatment<br>- Time since Diagnosis= 15.2 years (SD = 7.19, range: NA)<br>- Stage: NA<br>- Year of survivor: NA	– Rural survivors reported lower mean SF-36 Physical Component scores compared to non-rural survivors (P< .001). However, there were no significant differences in Mental Component between rural and non-rural survivors.<br>– Rural residence was significantly associated with SF-36 Physical Component Score (t = 2.30, P = .0217) and lower SF-36 Physical Function Subdomain (t = 2.53, P = .0116)	Short Form-36 Questionnaire (SF-36) version 2.0	- Area of the residence	- Results indicate that rural residence is associated with lower SF-36 PCS scores, reflecting poorer physical well-being.<br>- Future research should further explore these relationships to better target interventions and resources for rural cancer survivors.
**Study 35/ 100%**	**(Wasse et al., 2025)**<br>2025<br>France<br>Postal survey	- Non-Hodgkin lymphoma (NHL) [DLBCL = 239 (48%), FL 254 (52%)]<br>- n=493<br>- Age range (58-75), (65±13), median = 67	- To compare the HRQoL of NHL survivors to that of the general French population<br>- To identify factors impacting HRQoL in NHL survivors.	- Chemotherapy (R-CHOP) 439 (93%)<br>- Time since diagnosis 8.29±2.57<br>- Stage I-II = 155(35%),	- NHL survivors reported significantly lower HRQoL than the general French population except in terms of bodily pain (p<0.01).	The 12-item Short-Form health survey (SF-12)	- Personcal characteristics<br>- Clinical characteristics<br>- Psychological characteristics<br>- Sexual health<br>- Economic status	- Integrated Psychosocial Care: Routine screening and management of anxiety and depression must be integrated into survivorship care

Study No./ Quality Assessment	References, Published year, Country, Settings	Target population, Study design, Sample size (n), Age of participants (mean ±SD), Gender	Purpose of Study	Treatment, Time since diagnosis, Stage, Year of Survivor	Main Outcome (Factor Impacting Quality of Life, QoL) Only significant (B, OR, P-value)	QoL Measurement	Themes (factor influencing QoL)	Implications/ Suggestions
		- Male 274(56%), female 219(44%)		stage III-IV = 291 (65%)<br>- Year of survivors: N/A	**Factors significantly associated with lower HRQoL (Negative Impact):**<br>- Comorbidities: Strong negative association across all domains, e.g., General Health (B=-9.7, p<0.0001).<br>- Depression: Strong negative association across all domains, e.g., General Health (B=-13.9, p<0.0001).<br>- Socioeconomic Deprivation (EPICES): Negative association with General Health (B=-7.0, p=0.001) and Physical Functioning (B=-13.4, p=0.0006).<br>- Sexual Satisfaction Problems: Associated with lower Role Physical (B=-0.2, p=0.001) and Role Emotional (B=-0.2, p=0.0006).<br>- Age: Increasing age negatively impacts General Health (B=-0.2, p=0.0001).<br>- BMI (Obesity ≥30): Negatively affects Physical Functioning (B=-9.6, p=0.008).			- Sexual Health Dialogue: Clinicians should proactively initiate discussions on sexual satisfaction, moving beyond functional assessments to address unaddressed concerns that significantly impair role functioning and emotional well-being<br>- Targeted Equity Interventions: Survivorship strategies require stratification to support vulnerable subgroups—specifically those with economic status (financial strain) and multimorbidity—to mitigate disparities in long-term outcomes<br>- Lifestyle Management: Interventions focusing on weight management and physical health are critical, as obesity is an independent risk factor for deteriorated physical functioning

Study No./ Quality Assessment	References, Published year, Country, Settings	Target population, Study design, Sample size (n), Age of participants (mean ±SD), Gender	Purpose of Study	Treatment, Time since diagnosis, Stage, Year of Survivor	Main Outcome (Factor Impacting Quality of Life, QoL) Only significant (B, OR, P-value)	QoL Measurement	Themes (factor influencing QoL)	Implications/ Suggestions
					**Factors significantly associated with higher HRQoL (Positive Impact):**<br>- Male Gender: Associated with better General Health (B=4.2, p=0.01) and less Bodily Pain (B=6.8, p=0.007).<br>- Time Since Diagnosis: Longer time associated with improved Social Functioning (B=1.2, p=0.009).<br>- Income: Higher income (>€5000) associated with better Mental Health (B=15.7, p=0.001).			in long-term survivors.
**Study 36/ 100%**	**(Olsson et al., 2024)**<br>2024<br>Sweden<br>Postal survey	- Diffuse large B-cell lymphoma survivors<br>- Cross-sectional study<br>- n=257<br>- Mean age and range→70.0(10.4), range =47-92<br>- Male = 144 (56%), female = 113 (44%)	- To examine the association of sexuality, body image and health-related quality of life among diffuse large B-cell lymphoma	- Chemotherapy (248: 99%), Immunotherapy (52: 20%), Radiotherapy (63: 25%), Other (22: 9%)<br>- Time since diagnosis→22.6 months (9.7) range (≤ 6 months - ≥ 37 months)<br>- Year of survivor: N/A	Finding based on the multiple regression model in this study (R2 = 39.6%) : factors significantly associated with global HRQoL<br>- Sexual relation has positively associated with HRQoL (B = 5.925, p=0.029)<br>- Body image has negative associated with HRQoL (B = -1.141, p=<.001)<br>- Comorbidities have negative associated with	The EORTC QLQ-C30 (ver.3)	- Clinical characteristic<br>- Psychological concern (body image)<br>- Sexual health	- Interventions should prioritize the relational dimensions of sexuality (intimacy, communication) over functional metrics, as these are stronger predictors of HRQoL in this population.<br>- Body Image Assessment: Routine screening for negative body image is warranted even in the absence of visible disfigurement

**Study No./ Quality Assessment**	**References, Published year, Country, Settings**	**Target population, Study design, Sample size (n), Age of participants (mean ±SD), Gender**	**Purpose of Study**	**Treatment, Time since diagnosis, Stage, Year of Survivor**	**Main Outcome (Factor Impacting Quality of Life, QoL)** Only significant (B, OR, P-value)	**QoL Measurement**	**Themes (factor influencing QoL)**	**Implications/ Suggestions**
					HRQoL (B=-11.143, p=<.001)			- Enhance nurse-led survivorship care by integrating specific competencies for discussing sensitive topics to address long-term psychosocial well-being.
